# Glutaryl-CoA Dehydrogenase Misfolding in Glutaric Acidemia Type 1

**DOI:** 10.3390/ijms241713158

**Published:** 2023-08-24

**Authors:** Madalena Barroso, Marcus Gertzen, Alexandra F. Puchwein-Schwepcke, Heike Preisler, Andreas Sturm, Dunja D. Reiss, Marta K. Danecka, Ania C. Muntau, Søren W. Gersting

**Affiliations:** 1 University Children’s Research, UCR@Kinder-UKE, University Medical Center Hamburg-Eppendorf, 20246 Hamburg, Germany; m.barroso@uke.de (M.B.); m.danecka@uke.de (M.K.D.); muntau@uke.de (A.C.M.); 2 Department of Molecular Pediatrics, Dr. von Hauner Children’s Hospital, Ludwig-Maximilians-University, 80337 Munich, Germany; marcus.gertzen@bkh-augsburg.de (M.G.); alexandra.puchwein@ukbb.ch (A.F.P.-S.); heike.preisler@med.uni-muenchen.de (H.P.); andreas.sturm@kssg.ch (A.S.); dunja.reiss@med.uni-muenchen.de (D.D.R.); 3 Psychiatry and Psychotherapy, Faculty of Medicine, University of Augsburg, 86156 Augsburg, Germany; 4 Department of Pediatric Neurology and Developmental Medicine, University of Basel Children’s Hospital, 4056 Basel, Switzerland; 5 Medizinische Klinik und Poliklinik IV, Klinikum der Universität München, 81377 Munich, Germany; 6 University Children’s Hospital, University Medical Center Hamburg-Eppendorf, 20246 Hamburg, Germany

**Keywords:** glutaric aciduria, GA1, GCDH, protein folding, organic aciduria

## Abstract

Glutaric acidemia type 1 (GA1) is a neurotoxic metabolic disorder due to glutaryl-CoA dehydrogenase (GCDH) deficiency. The high number of missense variants associated with the disease and their impact on GCDH activity suggest that disturbed protein conformation can affect the biochemical phenotype. We aimed to elucidate the molecular basis of protein loss of function in GA1 by performing a parallel analysis in a large panel of *GCDH* missense variants using different biochemical and biophysical methodologies. Thirteen GCDH variants were investigated in regard to protein stability, hydrophobicity, oligomerization, aggregation, and activity. An altered oligomerization, loss of protein stability and solubility, as well as an augmented susceptibility to aggregation were observed. GA1 variants led to a loss of enzymatic activity, particularly when present at the N-terminal domain. The reduced cellular activity was associated with loss of tetramerization. Our results also suggest a correlation between variant sequence location and cellular protein stability (*p* < 0.05), with a more pronounced loss of protein observed with variant proximity to the N-terminus. The broad panel of variant-mediated conformational changes of the GCDH protein supports the classification of GA1 as a protein-misfolding disorder. This work supports research toward new therapeutic strategies that target this molecular disease phenotype.

## 1. Introduction

Glutaric acidemia type 1 (GA1, OMIM #231670) is an autosomal recessive inborn error of lysine, hydroxylysine, and tryptophan metabolism due to deficiency of glutaryl-CoA dehydrogenase (GCDH) [1]. It is one of the most prevalent organic acidurias, with an estimated worldwide frequency of about 1 in every 100,000 births [2]. GCDH is a mitochondrial flavoprotein enzyme responsible for the dehydrogenation and decarboxylation of glutaryl-CoA to crotonyl-CoA and carbon dioxide [3]. Reduced GCDH activity results in the toxic accumulation of glutaryl-CoA and its dicarboxylic derivatives, namely glutaric acid and 3-hydroxyglutarate (Supplementary Materials, Figure S1) [1]. This leads to a complex phenotypic spectrum of neurologic complications that ranges from macrocephaly and frontotemporal atrophy at birth to encephalopathic crises prompting severe dystonic movement disorders or even death [4].

Two groups of GA1 patients with different biochemical severity are known: patients with high levels of glutaric acid in the urine (≥100 mmol/mol creatinine; high excretors, HE) and those with low levels (<100 mmol/mol creatinine; low excretors, LE) [5]. Urinary glutaric acid was shown to inversely correlate with GCDH residual enzyme activity [5]. HE patients typically show activities below 5%, whereas LE patients present up to 30% GCDH residual activity [3,6]. Moreover, it was demonstrated by in silico score modeling that highly pathogenic variants are significantly correlated with lower residual enzyme activity [7]. Despite these associations, no clear correlation between the clinical phenotype and genotype has been established [7,8,9,10].

Inclusion of GA1 into newborn screening programs has impressively changed the course of the disease. Early diagnosis allows for early treatment implementation and has drastically improved patient clinical outcomes by reducing acute encephalopathic crises and mortality [8]. The current GA1 therapy relies on a low-lysine diet to avoid the accumulation of neurotoxic metabolites accompanied by supplementation of a lysine-free, tryptophan-reduced amino acid mixture and treatment with l-carnitine to support the urinary excretion of toxic organic acids [5,11]. Patients with intercurrent infections are often hospitalized to receive a glucose infusion to avoid catabolism. Overall, the treatment is symptomatic; it is extremely burdensome and does not address the molecular phenotype. In addition, despite early treatment, 15–23% of patients still experience an encephalopathic crisis [12,13]. The burdensome long-term diet contributes to low adherence to treatment recommendations in about 32% of patients, which is associated with worse neurologic outcomes and increased mortality [8]. Moreover, patients identified through newborn screening may still develop fine motor and articulation deficits despite strict dietary treatment [14], and early treatment also proves inefficient in preventing renal complications associated with GA1 [8]. The considerable proportion of GA1 patients for which the current treatment remains insufficient and/or challenging supports the need for alternative therapeutic strategies. The elucidation of the underlying disease mechanism is, therefore, the prerequisite for developing new and effective therapies. Namely, understanding the impact of the genetic variants on enzyme function and biochemical outcome would allow for genotype-specific approaches.

Currently, over 350 variants in the *GCDH* gene have been published and listed in the *Human Gene Mutation Database*, of which more than 75% are missense variants (>245 disease-causing variants) distributed throughout the protein (www.hgmd.cf.ac.uk; accessed on 9 May 2023). In addition, a recent extensive literature collection by Schuurmans et al. identified 421 different GCDH pathogenic variants [7]. *GCDH* missense variants have been shown to impair catalytic function, substrate and co-factor binding, mitochondrial stability, and oligomerization, resulting in a loss of enzymatic function [15,16,17]. Several GA1-associated variants have been biochemically characterized using different research approaches, i.e., based on their origin, distribution, and molecular phenotype [3,15,16,17,18]. For example, variants R402W (c.1204C > T), A293T (c.877G > A), and A421V (c.1262C > T) are associated with low enzymatic activity and HE phenotypes, which are highly prevalent in specific populations [19]. Other common variants in particular populations include E365K (c.1093G > A) and E414K (c.1240G > A) [19]. R402W is the most common variant in people from a Caucasian background, accounting for 10–20% of alleles [20,21]. Variants such as R227P (c.680G > C) and V400M (c.1198G > A) are associated with LE and are also prevalent in closed communities [22,23].

We performed a parallel analysis of protein stability, hydrophobicity, oligomerization, aggregation, and enzyme activity in a large panel of GCDH missense variants using different biochemical and biophysical methodologies, including blue-native PAGE and Western blotting, size-exclusion chromatography, thermal shift assay, right-angle light scattering, and enzymatic assays. Our primary objectives were to characterize the molecular phenotypes associated with GCDH variants, to establish inter-variant comparison, and to understand how structural alterations can determine the biochemical phenotype. Furthermore, our study explored the potential classification of GA1 as a protein-misfolding disorder. The insights gained from this research will hopefully support the research and development of innovative therapeutic strategies specifically targeting this molecular disease phenotype.

## 2. Results

### 2.1. Selection and Mapping of GCDH Variants on the Crystallographic Structure

**Figure 1:**
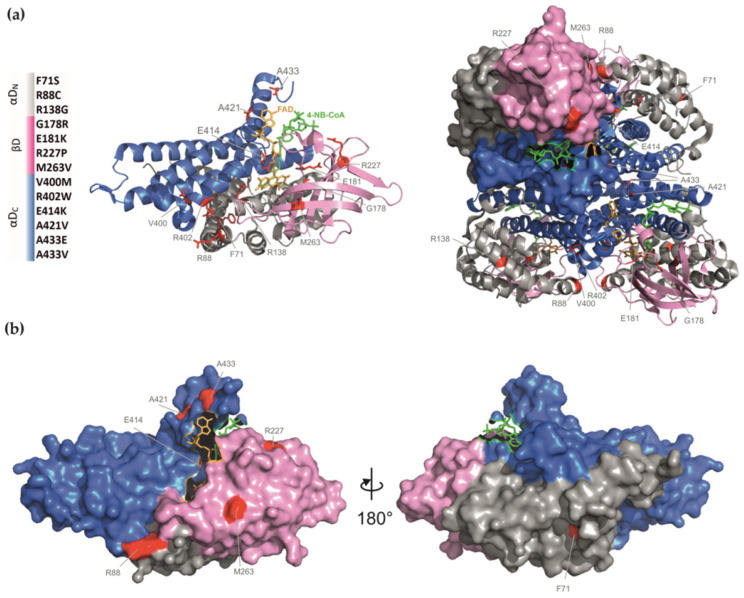
Human glutaryl-CoA dehydrogenase structure (PDB reference 1SIQ [43]): (**a**) Ribbon diagram of GCDH monomer (**left**) and tetramer (**right**) with labeling of the studied mutated residues, represented with red sticks. GCDH tetramer diagram includes a highlighted monomer with surface representation. Protein domains are colored in grey, pink, and blue for the N-terminal alpha-helical domain (αDN), the middle beta-sheet domain (βD), and the C-terminal alpha-helical domain (αDC), respectively. Color coding of the domains and the location of the studied variants are shown on the left. Co-factor and substrate analog binding are shown. The substrate analog 4-nitro butyryl-CoA (green) binds with the thioester moiety to the binding pocket composed of the αDN, the flavin group of the co-factor (FAD), and a loop of the αDC (E414). The adenosine–pantetheine moiety is located at the junction of αDN and αDC terminal domains. (**b**) Surface representations (180°-rotated) of the GCDH monomer.

Thirteen *GCDH* missense variants (Figure 1) were selected to analyze the impact on the loss-of-function phenotype associated with GA1. A broad range of variants was chosen based on several parameters: (1) allele frequency, including most known variants prevalent in particular populations; (2) sequence location, covering different protein domains; (3) associated biochemical phenotype (high and low excretors); and (4) molecular phenotype information available, including understudied and previously characterized variants. Conformational disorders are often associated with a plethora of missense mutations that span the entire protein sequence, a pattern that is evident in GA1. Protein misfolding is a common disease mechanism associated with genetic variation and presents a specific target for the development of new therapies. We selected a diverse set of variants to provide a comprehensive and representative sample, aiming to gain a deeper understanding of their impact on protein structure and misfolding.

The *GCDH* gene encodes the 438-amino-acid GCDH protein precursor form. The first 44 N-terminal residues are cleaved upon mitochondrial import, and the functional GCDH homotetramer comprises four 43 kDa subunits [15]. One protein monomer in the native conformation of the GCDH tetramer presents an ellipsoid structure (Figure 1). It contains three structural domains, namely an N-terminal alpha-helical domain (αD_N_, comprising the first six helices), a middle domain formed by two stacked beta sheets (βD, seven β-strands), and a C-terminal alpha-helical domain (αD_C_, last five helices). Three of the selected variants in this study map to the αD_N_ domain: F71S (c.212T > C), R88C (c.262C > T), and R138G (c.412A > G); four to the βD domain: G178R (c.532G > A), E181K (c.541G > A), R227P, and M263V (c.787A > G); and the remaining variants map to the αD_C_ domain: V400M, R402W, E414K, A421V, A433E (c.1298C > A), and A433V (c.1298C > T). Only residues R138, E181, and E414 are integral to structural moieties forming the substrate binding cleft and the catalytic center. All other residues map outside the structural parts associated with enzyme function. The side chains of most of the residues are partially (E414, R402, and E181) or completely (F71, R88, R227, M263, A421, and A433) oriented towards the protein surface [15]. There are several reports detailing the theoretical potential structural alterations stemming from amino acid substitutions in GCDH, encompassing different variants explored in this study and others [3,15,20,24].

The F71S mutation was first reported in a single homozygous patient [25], and to the best of our knowledge, no other studies have been conducted on this variant. In the same protein domain, the R88C allele has been described with a relatively high frequency (4%) [4,9,21] and found in both homozygotes and heterozygotes [9,21]. The R138G variant was described for the first time in 1998 [26], but no further reports on patients carrying this mutation exist to our knowledge. GCDH G178R, R227P, and M263V have been reported in patients from different populations as homozygotes or compound heterozygotes [9,25,27,28,29,30,31,32,33,34]. E181K was found in one homozygous patient [25]. V400M and R402W are both found in homozygous [17,35] and heterozygous [25] patients and represent the most common GCDH variants in the αDC domain [9,25]. V400M is strongly associated with a LE phenotype and high residual activity in heterozygotes [36,37], while R402W is one of the most severe common variants of GCDH [15,22]. GCDH activities as high as for M263V homozygotes (30% of control activity) have been reported for compound heterozygotes V400M and R227P [15,30]. Variants E414K, A421V, and A433V were also found in homozygous and compound heterozygous patients [4,9,17,24,25,33,38,39,40,41,42]. A single patient carrying the GCDH A433E variant has been described so far, in heterozygosity with R94L, with residual activity below 0.5% in cultured fibroblasts [37].

### 2.2. Impact of GA1-Associated Variants on GCDH Enzymatic Activity

Compared to the wild type (WT), each GCDH variant resulted in a loss of enzyme activity in both the cell-based and purified protein assays (Table 1). All variants distinctively compromised the enzymatic activity of purified GCDH independent of their sequence and domain location. GCDH tetramers of variants F71S, G178R, E181K, R227P, and R402W were not tested because these could not be purified in sufficient amounts for the activity assay. Variants M263V, V400M, and A433V, mapping to the βD and αD_C_ domains, displayed the highest level of residual GCDH activity (>18% of WT levels). All the other tested variants presented activity levels below 6% of WT GCDH activity. 

Most of the variants rendered an inactive enzyme in the cell-based assay. Variants G178R, E414K, and A433V presented an activity level below 1%, while M263V, V400M, and A421V activities were ≥10% that of WT. Notably, recombinant purified GCDH variants M263V and A433V displayed a significant residual activity, which was almost completely lost in vivo (cell-based assay). A421V, a variant associated with no activity in patient cells (Supplementary Materials, Table S1), was the only variant showing higher residual activity in the cell-based compared to the purified protein assay. In addition, the absence of residual activity induced by variants in the N-terminal alpha-helix domain suggests that GCDH cellular activity may be especially compromised when variants occur in this region of the protein. The relative GCDH activity measured in lysates of COS-7 cells overexpressing different protein variants was similar to that determined in patient cells carrying the genotype in homozygosity (Table 1 and Supplementary Materials, Table S1).

### 2.3. GCDH Missense Variants Lead to Loss of Protein Stability and Impaired Tetramerization

**Figure 2:**
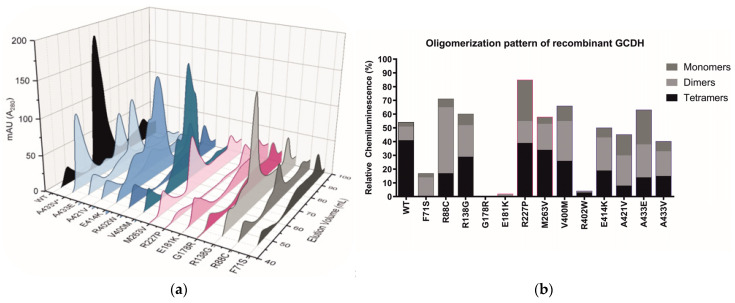
Size-exclusion chromatography of recombinantly expressed GCDH protein variants and oligomerization pattern of recombinant GCDH: (**a**) 3D representation of the elution profiles of 14 protein variants purified following a 2-step affinity and size-exclusion chromatography. Wild-type GCDH (black) was mainly eluted in the tetrameric form. All other variants showed a disturbed pattern of oligomerization with a higher proportion of monomers, dimers, and/or aggregates. Elution profiles are colored in grey, pink, and blue for the variants located in the N-terminal alpha-helical domain (αDN), the middle beta-sheet domain (βD), and the C-terminal alpha-helical domain (αDC), respectively. (**b**) Different GCDH protein variants were purified by affinity chromatography. Percentages of the different protein oligomeric states, determined by BN-PAGE followed by Western blotting, are represented as stacked bars for each protein variant. Percentages were calculated as the ratio of the intensity of the band corresponding to that oligomeric state (monomer, dimer, and tetramer) to the total intensity of the bands (100%).

To address the impact of the variants on the protein tetramer stability and oligomerization state, we assessed the oligomerization profile of purified WT and variant GCDH proteins. This was achieved by affinity chromatography followed by size-exclusion chromatography (Figure 2a) or blue-native PAGE (BN-PAGE) and Western blot (Figure 2b). The GCDH functional unit is a homotetramer of 173.2 kDa [17]. Notably, an increase of smaller oligomers (dimers 86.6 kDa and monomers 43.3 kDa) indicates a loss of protein stability. Our results show an altered GCDH oligomerization pattern of the GCDH variants compared to WT, implying an effect of the variants on the folding/conformation of the protein. Size-exclusion chromatography allowed the separation of the different protein oligomers, including higher oligomers or aggregates (elution volume between 45–55 mL), tetramers (65–75 mL), dimers (75–85 mL), and monomers (85–95 mL) (Figure 2a). While the pre-filtration step helped remove any major aggregates, the variant A433V clearly showed a propensity for forming large oligomeric complexes, as indicated by the peak at 50 mL (Figure 2a). Of note, A433V could only be purified in the presence of an alternative buffer (see Section 4). Chromatograms of variants F71S, G178R, E181K, and R402W revealed a loss of tetrameric units, which was confirmed by gel separation (Figure 2). Interestingly, variant R227P could not be purified by size-exclusion chromatography, but the presence of tetramers and other oligomers was visible on BN-PAGE (Figure 2b). Variants R138G, M263V, and V400M, which consistently exhibited the highest relative number of tetramers in both methodological approaches, also showed higher residual activity in the two activity assays (Table 1), emphasizing the importance of tetramerization for a functional enzyme unit. Moreover, the relative number of tetrameric units for recombinant GCDH (rGCDH) showed a significant correlation to the enzyme activity of rGCDH (*p* = 0.02) (Supplementary Materials Table S2).

### 2.4. Variant Location Correlates with Cellular GCDH Levels

**Figure 3:**
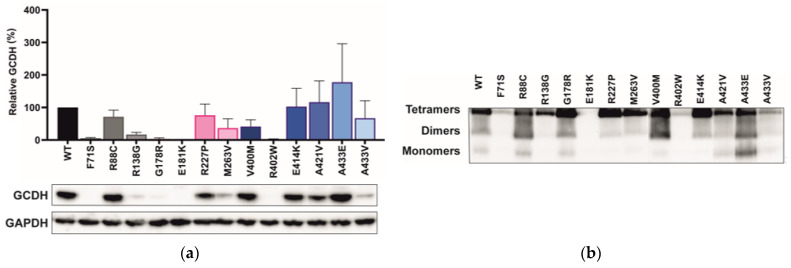
GCDH mammalian cell protein levels (**a**) and oligomerization pattern of cellular GCDH (**b**): (**a**) GCDH protein levels were determined by SDS-PAGE followed by Western blotting. Summary densitometry measurements (*n* = 3), corrected for GAPDH intensity and normalized to wild type, are shown (**above**) together with a representative immunoblot (**below**). Data for each mutant are colored in grey, pink, and blue for their correspondent location at the N-terminal alpha-helical domain (αDN), the middle beta-sheet domain (βD), or the C-terminal alpha-helical domain (αDC), respectively; (**b**) lysates of mammalian cells expressing different GCDH protein variants were used for BN-PAGE followed by Western blotting. Chemiluminescence imaging of the blots is shown.

Cellular protein levels following transient expression of the GCDH variants in COS-7 cells were determined by SDS-PAGE followed by Western blot analysis (Figure 3a). The GCDH protein oligomerization status in the cell lysates was monitored by BN-PAGE followed by Western blot detection (Figure 3b). Most GCDH variants presented a loss of protein in comparison to WT, with a severe loss (<20% of WT) observed for five variants (F71S, R138G, G178R, E181K, and R402W). Various variants close to the αD_C_ domain (E414K, A421V, and A433E) showed a similar or higher protein level than WT. The variant sequence location of our panel of GCDH variants showed a significant correlation with the cellular protein level of GCDH (*p* = 0.05). Cellular GCDH levels decreased with proximity of the variant to the N-terminus (Supplementary Materials, Table S2). Low cellular GCDH levels may contribute to the severe loss of activity observed for variants F71S, G178R, E181K, and R402W. Nevertheless, several variants (R88C, R227P, E414K, A421V, A433E, and A433V) with significant protein levels (36–178% of WT) showed a lack of residual enzyme activity. 

The oligomerization pattern of the different variants expressed in COS-7 cells and analyzed by Western blot is shown in Figure 3b. Intracellular expression of the GCDH variants resulted in disturbed oligomerization in the alpha-helix domains (R88C, V400M, A421V, and A433E), as observed for the purified rGCDH variants (Figure 2b). The impaired tetramerization of the variants whose protein levels were not significantly altered compared to WT (i.e., R88C, A421V, and A433E) may contribute to the loss of enzymatic activity observed. Notably, the intracellular protein levels generally align with those of the overall expression observed in the BN-PAGE blots.

### 2.5. The GCDH Misfolding Phenotype Is Confirmed by Increased Hydrophobicity, Aggregation, and Loss of Thermal Stability

**Figure 4:**
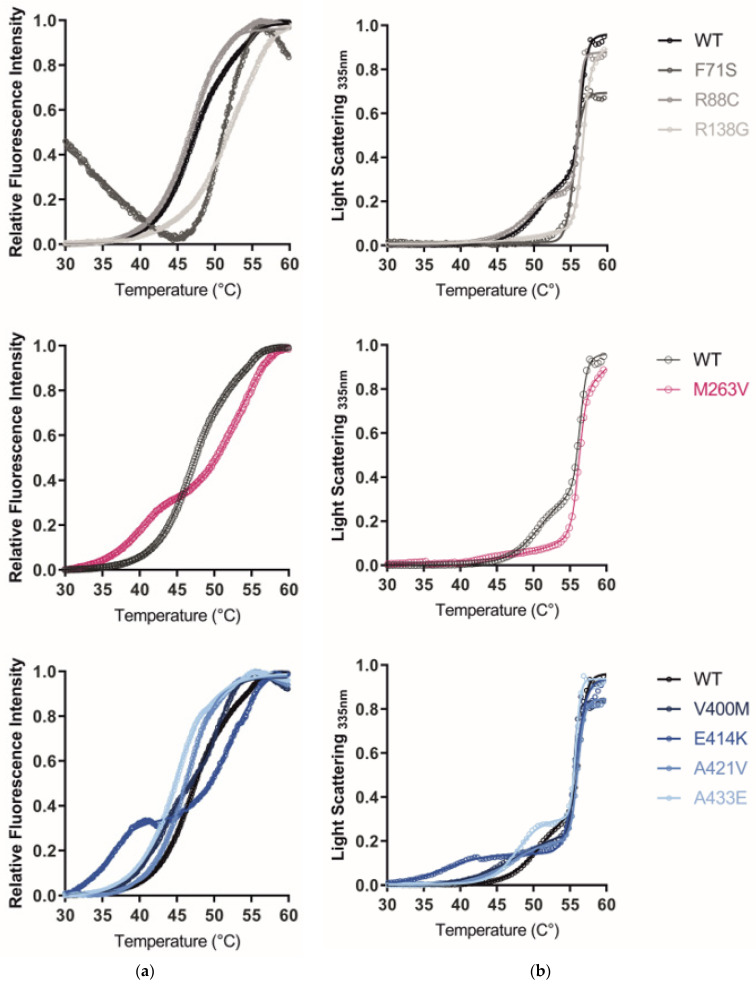
GCDH thermal denaturation and aggregation profiles: (**a**) Purified recombinant GCDH accelerated thermal denaturation for most studied variants. The thermal unfolding of wild type and variants was monitored by thermal shift assay using SYPRO orange. Normalized fluorescence intensity is plotted as a function of increasing temperatures (circles) together with the fit biphasic model (lines). (**b**) Aggregation profiles of recombinant GCDH. Variant GCDH proteins show accelerated aggregation with higher temperatures. Aggregation profiles were monitored by right-angle light scattering. Turbidity (dynode voltage) melting curves recorded at 335 nm demonstrate the heat-induced increase in the particle size due to protein aggregation. (**a**,**b**) Each graph includes the denaturation/aggregation profile of wild type and variants located in a specific domain: N-terminal alpha-helical domain (**top**), the middle beta-sheet domain (**middle**), or the C-terminal alpha-helical domain (**bottom**).

Altered protein conformation by the exposure of structural hydrophobic residues was monitored by following the fluorescence emitted by SYPRO orange binding. This molecular dye exhibits very low fluorescence in its unbound state in aqueous solution, as its molecules are predominantly quenched. However, when interacting with protein hydrophobic residues, fluorescence increases. Except for A433V, all purified GCDH variants showed elevated fluorescence at 25 °C in comparison to WT (*p* < 0.001; Table 2), indicating increased hydrophobicity. Namely, variants F71S, R138G, V400M, and A421V rendered the highest fluorescence values (over 3.4-fold increase compared to WT). In order to evaluate protein stability under thermal stress, a thermal shift assay was performed. Figure 4a depicts the protein denaturation profiles of each purified GCDH variant. Each variant exhibited a different protein denaturation profile. For most of the variant proteins, a biphasic model was used to fit the fluorescence data acquired from 30–60 °C, and the two curve-transition points (Tm_1/2_ and Tm_2/3_) were used to compare protein stability (Table 2); an exception was variant F71S, where a biphasic fitting was not possible, and a single transition point was calculated. The observed right shift of the F71S denaturation curve requires careful consideration. The initial fluorescence of F71S (25–30 °C) was aberrantly high compared to WT and other variants, suggesting a significantly perturbed protein structure at the start of the experiment, with high exposure of hydrophobic residues [44,45]. In order to further clarify the effect of this variant on protein thermal stability, alternative methods are required, such as circular dichroism (CD) or Fourier-transform infrared spectroscopy (FTIR) analysis. A loss of thermal stability was observed for all other variants, with the αD_C_ domain being the most affected. A distinct two-step denaturation process was observed for the variants M263V and E414K, where the more pronounced transition phases may reflect a similar denaturation pattern.

Right-angle light scattering (RALS) measures the intensity of scattered light by a protein in solution at a 90° angle. The amount of scattered light increases with larger particles in solution, allowing for protein aggregation monitoring. The overall turbidity at 25 °C (Table 2) was used as an indicator of aggregation. F71S showed the highest turbidity (7.5-fold higher than WT), and all variants mapping to the alpha-helical domain showed increased values. Aggregation profiles upon thermal stress are shown in Figure 4b. The amount of scattered light over increasing temperatures could be modeled using a biphasic fitting, indicating a two-step aggregation. Compared to WT, the first transition point was decreased for all variants, indicating an increased propensity for aggregation. At the second transition point, where higher aggregates are formed, the temperature remained around 56 °C (Tm_2/3_) for all GCDH variants (Table 2). As in the thermal shift assay, variants mapping to the αD_C_ domain had a pronounced effect on RALS, while most variants mapping to the αD_N_ domain and the βD domain showed lower levels of aggregates, suggesting increased resistance of these proteins against aggregation upon thermal stress (Figure 4b). 

### 2.6. Protein Expression at Lower Temperatures Rescues Mutant GCDH Solubility

**Figure 5:**
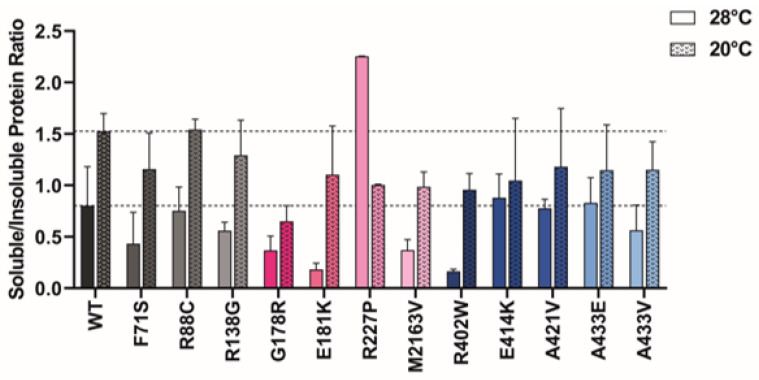
Temperature effect on recombinant GCDH solubility. The solubility of wild type and variant GCDH proteins was analyzed after recombinant expression induction with isopropylthio-β-D-galactoside and riboflavin at temperatures of 20 and 28 °C. Ratios were calculated based on the chemiluminescence intensities of Western blots following SDS-PAGE of recombinantly expressed soluble and insoluble lysate extracts. Data for each mutant are colored in grey, pink, and blue for their correspondent location at the N-terminal alpha-helical domain (αDN), the middle beta-sheet domain (βD), or the C-terminal alpha-helical domain (αDC), respectively.

Expression of recombinant human GCDH could be achieved at 28 °C. At this temperature, protein solubility was estimated based on the amount of protein detected in solution or present in the insoluble pellet fraction, using SDS-PAGE followed by Coomassie staining. The ratio of soluble to insoluble protein was calculated (Figure 5). A loss of solubility was evident for most GCDH variants studied. Variants F71S, G178R, E181K, M2613V, and R402W had soluble/insoluble ratios lower than 0.5%. GCDH variants mapping to the middle βD domain were the most affected, while variants in the αD_C_ domain were least affected compared to WT, i.e., presented the highest ratios. No direct correlation with aggregation profiles could be established. However, tetramerization levels significantly correlated with the solubility ratio at 28 °C (*p* = 0.02), suggesting an association between decreased solubility and loss of tetramers.

Considering the relevance of temperature in GA1, we assessed whether a decrease in temperature during protein expression could rescue protein solubility. Protein expression at 20 °C significantly increased protein solubility for most GCDH variants. R227P was the only exception, with a higher temperature showing an increased soluble-to-insoluble-protein ratio (Figure 5). R402W presented a striking 6-fold increase in the soluble to insoluble ratio. However, this was the largest increase in the αD_C_ domain, with other variants in this region showing a ≤2-fold increase. αD_N_ and βD domains especially benefited from expression at lower temperatures, with the soluble to insoluble ratio increasing between 1.8- to 6-fold for variants in these domains (except for R227P).

## 3. Discussion

This paper presents novel genotype–molecular phenotype correlations of the GCDH wild type and variant GCDH proteins arising from thirteen GCDH variants with a broad structural distribution and avoidance of structural hotspots. All selected variants mapping to the N-terminal alpha-helical domain (αDN) resulted in a complete loss of enzymatic activity when expressed in mammalian cells. Variants in the middle beta-sheet domain severely compromised recombinant protein expression and purification, whereas variants in the C-terminal alpha-helical domain (αDC) showed altered oligomerization profiles, namely decreased formation of tetramers. The simultaneous analysis of the GCDH variants enabled us to compare molecular features, explore novel variant–phenotype correlations, and understand how variant sequence location may be suggestive of specific protein misfolding features.

### 3.1. Enzyme Activity Findings

The variants analyzed in this study result in a loss of function of the GCDH enzyme, which is known to be associated with the patient’s biochemical phenotype [4]. Residual activity from seven of the variants studied was previously determined in patient-derived cells (Supplementary Materials, Table S1). The relative GCDH activity measured in lysates of COS-7 cells overexpressing different protein variants was similar to that determined in patient cells carrying the homozygous genotype. Relative enzymatic activity for R138G, M263V, R402W, and E414K in cell culture was consistent with previous studies using equivalent models [22]. M263V and V400M, known to be associated with LE phenotypes [2], showed the highest residual activity. The variant V400M exhibited the least reduction in activity when taking both assays into account. Accordingly, this variant is also the one with the most preserved cellular protein levels and tetrameric state, suggesting that it is one of the most stable variants. Nevertheless, it is known to disturb protein folding, showing a less compact structure and a loss of thermal stability [3], which is verified here. Previously, it was suggested that the instability of this variant may benefit from the interallelic complementation with R227P, where it can contribute for substrate binding and restored activity [3].

Overall, enzyme activity analyzed using purified rGCDH correlated with activity measured in cell lysates. However, differences were found when the two models were compared. For example, R138G and A433E variants showed a more significant loss of activity in vivo than in vitro. A reduction of intracellular protein stability, as observed for the variant R138G, potentially mediated by the intracellular proteostasis degradation machinery, could contribute to such disparity. In addition, the assay conditions used for testing the endogenous and purified enzymes differed. The assay used for purified protein monitors the electron transfer to an artificial electron acceptor and may not reflect the ability of the enzyme to form crotonyl-CoA intracellularly. The GCDH variant A433E may not be able to establish a protein–protein interaction with its endogenous electron acceptor protein (electron-transferring flavoprotein, ETF) [46] but may still be partially active in the presence of a small chemical electron acceptor. In fact, residue modification at the protein’s surface is more likely to impact this protein–protein interaction [46]; the A433 residue is oriented towards the protein surface, supporting this hypothesis. Nevertheless, additional studies are required to further assess the impact of the cellular proteostasis network or the methodologies used on the enzyme activity of the variants analyzed.

### 3.2. Tetramerization Findings

A previous study on GCDH αDC variants showed that variants in this region mainly affect subunit interaction and tetramerization, leading to impaired binding to flavin adenine dinucleotide (FAD) and substrate [47]. This observation concurs with our finding that variants in the αDC domain have altered oligomerization profiles, namely decreased formation of tetramers. Notably, GCDH monomers and dimers were previously found to be catalytically inactive [16]. In addition to R88C, V400M, A421V, and A433E, which showed an altered oligomerization pattern in cell lysates, variants R138G, R227P, and M263V also presented a loss of tetramerization when expressed recombinantly. The cellular tetramerization of these variants may be supported in vivo by endogenous eukaryotic chaperones. Increased hydrophobicity of GCDH variants further supports their conformational instability and may contribute to aggregate formation and loss of tetramers [48]. Variants in the αDC also rendered a marked decrease in thermal stability and increased protein aggregation, which could further contribute to the protein loss of function. Interestingly, the A433E variant, despite its increased hydrophobicity, did not show an increase in initial turbidity. However, under thermal stress, its propensity for aggregation became more evident, as indicated by a 3.5 °C decrease in the Tm_1/2_ value as determined by right-angle light scattering (RALS). This, along with the observed decrease in both thermal shift assay (TSA) melting transitions compared to WT, suggests a A433E-induced loss of GCDH thermal stability. These findings underscore the complex outcomes of genetic variants on protein conformation. The residue substitution can alter protein conformation and expose hydrophobic residues, which does not always lead to a clear increase in aggregation. However, a decrease in thermal stability is consistently observed. These observations highlight the significance of a comprehensive panel of biophysical tests, particularly the meticulous examination of thermal stability. This is especially important when considering a disorder where a patient’s elevated body temperature can precipitate an irreversible, devastating catabolic crises.

### 3.3. Variant Location Correlates with Cellular GCDH Levels

Notably, all variants in the αDN resulted in a complete loss of function of the enzyme expressed in mammalian cells. This activity loss was related to a severe loss of protein stability, as revealed by the decreased protein levels in GCDH variants in this domain. Busquets et al. previously suggested that, except for the first five, αDN residues are not directly involved in either substrate binding or oligomerization [25]. Therefore, decreased protein stability may play a major role in GCDH loss of function due to variants in the αDN. Interestingly, the R88C substitution was the only αDN variant studied that presented almost WT-like protein levels with compromised tetramerization. Previous studies have shown that unstable GCDH variants can still be transported into the mitochondria, which are degraded by poorly characterized mechanisms [15,24]. R88C impairs mitochondrial morphology [24], and intra-mitochondrial protein degradation machinery may be compromised, resulting in the still-detectable non-functional variant. 

Variants in βD (G178R, E181K, R227P, and A293T) also compromised protein stability, resulting in low protein levels. In contrast, most αDC variants showed wild-type-like protein levels. The lack of PEST motifs in the C-terminal region supports a lower vulnerability of this domain to proteasomal degradation in comparison to the αDN and βD domains (https://emboss.bioinformatics.nl/cgi-bin/emboss/epestfind (accessed on 19 June 2021)) [49]. In addition to the structural disorder-associated PEST sequences, N-terminal arginine residues can signal proteins toward degradation [50,51]. The cleavage of the GCDH mitochondrial targeting sequence exposes an arginine residue, which could also contribute to preferential N-terminal degradation. 

### 3.4. The GCDH Misfolding Phenotype Is Confirmed by Increased Hydrophobicity, Aggregation, and Loss of Thermal Stability

Disturbed protein conformation can result in the exposure of usually inaccessible hydrophobic residues [52]. In vitro analysis of purified recombinant protein in the presence of SYPRO orange showed that all GCDH variants except for A433V display increased hydrophobicity. F71S, shown to compromise tetramer formation, resulted in low stability with almost undetectable protein and a 3.7-fold increase in hydrophobicity level compared to the WT, supporting a drastic conformational change driven by this variant. The disruption of the salt bridge between the neighbor residue R72 and E129, suggested by Busquets et al. [25], could contribute to the observed conformational changes. In addition, most variants analyzed here presented a decrease in thermal stability and an increased propensity for aggregation. Namely, all αDC variants presented high susceptibility for these misfolding phenotypes. Interestingly, when following M263V and E414K denaturation profiles using thermal shift assays, both mutants showed distinct transition phases, which may reflect a similar region-specific biphasic denaturation. The biphasic unfolding behavior observed in certain variants could be attributed to an initial, faster unfolding of protein tetramers, followed by a subsequent unfolding of the remaining protein core, likely reflecting the instability induced by these variants in the oligomerization (αDC domain) and dimer–dimer interaction regions (βD domain). However, this hypothesis requires further experimental validation.

Considering the turbidity of recombinant protein in solution, a variant-associated increase in aggregation was observed for variants in both alpha-helical domains. A biphasic model was used to retrieve the transition midpoints that fit the aggregation profiles given by right-angle light scattering measurements following a temperature ramp. The first transition (Tm_1/2_) was decreased for all variants where the model was successfully applied, indicating an increased propensity for aggregation. Still, for variants F71S, R138G, M263V, and E414K, the biphasic model did not represent the obtained data. Interestingly, these variants showed a spike in fluorescence only at higher temperatures compared to the WT, suggesting that the conformational changes associated with these variants may confer resistance to aggregation at temperatures above 45 °C. However, the protein’s native state is already entirely compromised at this temperature. This is corroborated by the reduced solubility of F71S, R138G, and M263V compared to WT protein at 28 °C (Figure 5) and the heightened turbidity of the R138G, E414K, and particularly the F71S variants at 25 °C (Table 2).

### 3.5. Protein Expression at Lower Temperatures Rescues Variant GCDH

In contrast to previous findings, where authors found that bacterial extracts with recombinantly expressed GCDH variants grown at 25° C presented only 30% of activity when compared to those expressed at 37 °C [16], lower temperatures resulted in better yields of soluble GCDH variant proteins in our study. Several factors have been shown to contribute to increased protein stability and better protein folding in *E. coli* at lower temperatures, including favored hydrophobic interactions, avoidance of aggregation caused by higher temperatures [47], decreased propensity for the formation of inclusion bodies [53], inhibition of heat-shock proteases, and increased chaperone expression [47]. Interestingly, the R227P variant exhibited high solubility, which contrasts with the low yield observed during protein purification attempts. The lysates used for solubility tests were prepared in a potassium phosphate buffer, differing from the HEPES buffer used in size-exclusion purification. This discrepancy may suggest a loss of solubility when buffer conditions are altered. Therefore, the development of new purification protocols may prove beneficial for the successful purification of this specific variant.

The impact of fever in GA1 is well known as a key trigger for the encephalopathic crisis associated with disease severity [54] and may be potentially linked to temperature-dependent severe misfolding episodes. Given this clinical relevance, the potential benefit of temperature control studies on GA1 patients is worth considering.

Despite using a broad panel of variants, our study is limited by the selections made, and relevant information might have been obtained from analyzing other protein variants. Notably, the new correlations need further confirmation by deep characterization of a yet larger variant panel. Furthermore, we rely on a protein tag to express the recombinant GCDH, which improves the overall protein stability and contributes to the observed behavior of the studied proteins. Nevertheless, the proteins studied were expressed in the same conditions permitting reliable data comparison. 

Several of our assays rely on recombinant purified protein. The utilization of prokaryotic expression systems for human protein expression is limited by their inability to perform post-translational modifications, which may impact their conformation and function. Indeed, post-translational modifications such as glutarylation were described to occur in GCDH and significantly influence function. The interplay between these PTMs and the conformational changes induced by amino acid substitutions presents an intriguing avenue for future research. 

In the era of personalized medicine, understanding the implications of each genetic variation is crucial. Further research could benefit from a deeper delve into secondary structure analysis and independent domain studies, providing other insights into mutation impacts. As artificial intelligence progresses, the data from our study and equivalent others could be invaluable for training predictive models. These models could potentially expedite the identification of therapeutic options by predicting the impact of unexplored mutations.

## 4. Materials and Methods

### 4.1. DNA Constructs

The cDNA of human GCDH was cloned into the prokaryotic pMAL-c2X expression vector (New England Biolabs, Ipswich, MA, USA) encoding an N-terminal maltose-binding protein (MBP) and into the eukaryotic pEF DEST 51 V5 expression vector (Invitrogen, Waltham, MA, USA). GCDH mutants were generated using the QuikChange site-directed mutagenesis kit (Stratagene, San Diego, CA, USA). The authenticity of mutagenesis was verified by DNA sequencing.

### 4.2. Prokaryotic Expression and Purification

Human GCDH was expressed in *Escherichia coli* strain BL21 DE3 (Stratagene). Bacteria cells were grown to mid-exponential phase at 20 °C in LB medium (16 g/L tryptone (Oxoid, Basingstoke, UK), 5 g/L yeast (Oxoid, Basingstoke, UK), 2.5 g/L K_2_HPO_4_ (Merck, Darmstadt, Germany), and 0.1% glucose (B. Braun, Melsungen, Germany)) containing 100 µg/mL ampicillin. Overexpression of wild-type and variant GCDH anti-myelin basic protein fusion proteins was induced with 0.3 mM isopropylthio-β-D-galactoside with the addition of 1 µg/mL riboflavin (Sigma-Aldrich, St. Louis, MO, USA) at a post-induction temperature of 28 °C (or 20 °C). After 22 h of expression, samples were disrupted by sonification and, after centrifugation, split into soluble and insoluble (pellet) fractions. These samples were used for direct expression analysis based on electrophoretic separation and immunoblotting, or the soluble fraction was used for protein purification. All variants were purified with the same starting conditions. The entire soluble fraction collected was used for protein purification.

Recombinant proteins were purified at 4 °C using an ÄKTA purifier and ÄKTA express systems (GE Healthcare, Chicago, IL, USA). Briefly, the bacterial crude extract was loaded (30–50 mL) into an MBPTrap affinity chromatography column (GE Healthcare, Chicago, IL, USA) equilibrated with the lysate’s buffer, 10 mM potassium phosphate buffer (Merck, Darmstadt, Germany) containing 10% glycerol (Pharmacy of the LMU Klinikum München, Munich, Germany), 1 M DTT (Fluka, Charlotte, NC, USA), and one complete mini EDTA-free tablet (Protease Inhibitor Cocktail, Roche, Basel, Switzerland). Protein elution was achieved in the same buffer supplemented with 10 mM maltose. Affinity chromatography was followed by size-exclusion chromatography using a HiLoad 16/600 Superdex 200 column (GE Healthcare, Chicago, IL, USA) and 20 mM HEPES pH 7.0, 200 mM NaCl (with an exception for GCDH variant A433V, where the above-mentioned 10% glycerol 10 mM potassium phosphate buffer was used). All protein isolated during the first purification step (concentrated into 5–10 mL) was used for size-exclusion chromatography (SEC). The protein tetramer fraction was isolated and diluted following protein concentration determination by spectrophotometry at 280 nm. An equivalent molecular weight was assumed for the different variants. Samples were promptly aliquoted and frozen in liquid nitrogen. Protein aliquots were checked by blue-native polyacrylamide gel electrophoresis (BN-PAGE).

### 4.3. Cellular GCDH Expression in COS-7 Cells

COS-7 cells were cultured in RPMI 1640 medium with stable glutamine (Gibco, Waltham, MA, USA) supplemented with 10% fetal bovine serum and 1% antibiotic-antimycotic solution (Corning, Corning, NY, USA). Cells were cultured in 6-well plates for protein isolation or 96-well plates for enzyme activity. Transient expression of GCDH was achieved by transfection of GCDH-containing pEF-DEST51 using an Amaxa electroporation system (Lonza, Basel, Switzerland; 1 µg DNA/1Mio cells) and culture for 48 h. Cells were harvested by scraping and/or lysed by three freeze–thaw cycles in 20 mM Hepes, pH 7.0, and 200 mM NaCl buffer with one complete mini EDTA-free tablet (Protease Inhibitor Cocktail; Roche, Basel, Switzerland). The lysis buffer additionally contained 1% Triton X-100 for cells harvested for BN-PAGE. Total protein concentrations were determined using the Bradford assay.

### 4.4. Polyacrylamide Gel Electrophoresis and Immunoblotting

Sodium dodecyl-sulfate (SDS)-containing polyacrylamide gel electrophoresis (PAGE) was performed using pre-cast 10% Bis-Tris SDS-PAGE gels (Thermo Fisher, Waltham, MA, USA) as previously described [55] and used for direct staining with Coomassie Brilliant Blue R-250 (Bio-Rad) or immunoblotting (as described below). In addition, BN-PAGE was used to assess protein oligomerization. BN-PAGE was performed as described in [55]. Western blotting was then performed following semi-dry transfer. Immunoblotting was performed on nitrocellulose or PVDF membranes (GE Healthcare, Chicago, IL, USA) using primary anti-V5 (Invitrogen, Waltham, MA, USA, 1:5000 dilution), anti-MBP (New England Biolabs, Ipswich, MA, USA, 1:10,000), anti-GCDH (New England Biolabs, Ipswich, MA, USA, 1:1000) and anti-GAPDH (Meridian, Deerfield, IL, USA, 1:40,000, loading control) antibodies, and horseradish peroxidase-conjugated goat anti-mouse (Santa Cruz, Dallas, TX, USA, 1:10,000 dilution). Blots were visualized with Super Signal^®^ West Femto Maximum Sensitivity Substrate (Thermo Scientific, Waltham, MA, USA), and chemiluminescence was monitored and quantified with a DIANA III (Raytest, Liège, Belgium) or ChemiDoc MP (Biorad, Hercules, CA, USA) imaging systems and corresponding analysis software. 

### 4.5. Enzyme Activity Assays

Two independent assays were used to assess GCDH activity. The first assay measured the activity of purified, recombinant GCDH (rGCDH) as previously described [56], with minor modifications. Briefly, purified rGCDH was assayed at 25 °C in reaction mixture (potassium phosphate buffer (44.5 mM, pH 7.2) containing glutaryl-CoA (100 µM), FAD (100 µM), and ferrocenium hexafluorophosphate (200 µM)) for 5 min. Ferrocenium hexafluorophosphate was used as an artificial electron acceptor, and its reduction was monitored spectrophotometrically at 300 nm (Clariostar, BMG LABTECH, Ortenberg, Germany). The second assay (COS-7 cells) measured the cellular GCDH activity based on 3-hydroxy butyryl-CoA quantification by high-performance liquid chromatography (HPLC). Due to the fast conversion of crotonyl-CoA to 3-hydroxy butyryl-CoA in the presence of enoyl-CoA hydratase, slower GCDH activity was monitored indirectly upon incubation of cell lysates with 0.075 mM FAD, 0.3 mM ferrocenium hexafluorophosphate, and 0.01 mM glutaryl-CoA in 75 mM potassium phosphate buffer (pH 7.4) for 45 min at 37 °C. The reaction was stopped and neutralized as previously described [57]. Glutaryl-CoA, crotonyl-CoA, and 3-hydroxy butyryl-CoA determination were performed using reversed-phase (C18 column) HPLC in a Dionex U3000 system (Thermo Fisher, Waltham, MA, USA) with UV detection (260 nm). Chemical standards for all tested metabolites (Sigma-Aldrich, St. Louis, MO, USA) were included. 

### 4.6. Right-Angle Light Scattering (RALS)

RALS experiments were performed on a Cary Eclipse fluorescence spectrophotometer with a temperature-controlled Peltier multicell holder (Varian, Palo Alto, CA, USA). Samples contained 0.6 mg/mL protein diluted in 20 mM HEPES buffer, pH 7.0, and 200 mM NaCl. The increase in turbidity was monitored in the temperature range from 25 to 62 °C at a heating rate of 1 °C/min (excitation at 330 nm, emission at 335 nm; 5.0 nm slit widths). Results were normalized by defining the smallest and largest mean value in each dataset as 0 and 100%, respectively. Each dataset includes triplicate samples. Normalization, curve modeling, and transition midpoints (from biphasic fitting) were calculated using GraphPad, San Diego, CA, USA.

### 4.7. Thermal Shift Assays

For analysis of thermal stability, GCDH protein was diluted in 20 mM HEPES buffer, pH 7.0, and 200 mM NaCl and, in the presence of the hydrophobic dye SYPRO orange (Thermo Fisher, Waltham, MA, USA), exposed to a temperature ramp from 25 to 60 °C at a heating rate of 1 °C/min. Thermal incubation and fluorescence detection was performed using a 7900 HT Fast Real-Time PCR System (Applied Biosystems, Waltham, MA, USA). Transition midpoints (Tm) were calculated as for RALS. 

### 4.8. Structural Analysis

Structural analysis was based on the available PDB human GCDH crystallographic structure complexed with 4-nitro butyryl-CoA (PDB code: 1SIR). Model manipulation for variant analysis and generation of 3D images was performed using PyMOL (Schrödinger, Inc., New York, NY, USA).

### 4.9. Statistics

Results are shown as means ± standard error of the mean. Statistical analysis was performed on experiments repeated in three to four independent assays. Statistical comparisons between wild-type and variant proteins were performed using ANOVA analysis followed by pairwise post hoc comparisons with Dunnett’s test. Statistical Analysis was performed using GraphPad Prism (GraphPad, San Diego, CA, USA)

## 5. Conclusions

The GCDH variants studied highlight the relevance of different conformational changes impacting enzyme activity. Although some variants may irreversibly disrupt the GCDH active site (e.g., Y414C), several could strongly benefit from conformational stabilization. With growing evidence of associations between patient clinical outcomes, namely chronic kidney disease and white matter abnormalities, and the biochemical phenotype, improving GCDH activity could impact patient quality of life. Our results suggest that all variants studied result in conformational alterations compared to wild type. The characterization of the folding defects of GCDH variant proteins can help in the discovery of new therapeutical strategies for this disorder. The use of targeted pharmacological chaperones is an approach that has proved successful in other conformational disorders such as phenylketonuria, cystic fibrosis, and Fabry disease [58], and it is worth considering whether drug-development processes could benefit from currently available GA1 animal models [57,59]. In GA1, age in relation to brain and motor development stages is crucial in the course of this disease. Therefore, the development of safe and non-invasive therapies based on the pharmacological rescue of enzyme activity is of outstanding clinical relevance. Our results provide the basis to explore these therapeutic approaches for different patient genotypes.

## Figures and Tables

**Table 1:** GCDH enzyme activity.

Protein Variant		Relative Crotonyl-CoA Formation (%)	
		rGCDH Assay	COS-7 Cellular Assay
	WT	100.0 ± 3.0	98.9 ± 1.1
	F71S	-	n.d.
	R88C	0.9 ± 0.0 *	n.d.
	R138G	4.0 ± 0.2 *	n.d.
	G178R	-	0.8 ± 0.5 *
	E181K	-	n.d.
	R227P	-	n.d.
	M263V	30.6 ± 2.2 *	13.1 ± 1.4 *
	V400M	49.4 ± 1.3 *	33.3 ± 4.5 *
	R402W	-	n.d.
	E414K	1.2 ± 0.1 *	0.9 ± 1.8 *
	A421V	2.5 ± 0.4 *	9.7 ± 2.8 *
	A433E	5.6 ± 1.9 *	n.d.
	A433V	18.3 ± 0.6 *	0.2 ± 2.6 *

The protein domains are marked in grey, pink, and blue for easy association with the αDN, βD, and αDC domains, respectively. αDN, N-terminal alpha-helical domain; βD, beta-sheet domain; αDC, C-terminal alpha-helical domain; rGCDH, recombinant glutaryl-CoA dehydrogenase; n.d., not determined; WT, wild type. * *p* < 0.05 (in comparison to WT, ANOVA analysis was performed independently for each enzymatic assay: rGCDH and cellular assay).

**Table 2:** Hydrophobicity, unfolding, and aggregation profiles of GCDH.

Protein Variant		TSA			RALS		
		F.I._25°C_ (AU) Mean ± SEM	Tm_1/2_ (°C) Mean (CI 95%)	Tm_2/3_ (°C) Mean (CI 95%)	Turbidity_25°C_ (AU) Mean ± SEM	Tm_1/2_ (°C) Mean (CI 95%)	Tm_2/3_ (°C) Mean (CI 95%)
	WT	41.5 ± 0.8	47.1 (47.0–47.1)	54.8 (54.6–55.0)	21.8 ± 0.4	51.2 (49.3–54.1)	56.4 (56.2–56.6)
	F71S	154.1 ± 6.6 *	51.4 (model is unfit)	–	164.0 ± 2.1 *	–	–
	R88C	85.2 ± 2.8 *	39.2 (38.3–40.5)	47.0 (46.9–47.1)	39.9 ± 0.4 *	49.5 (49.1–50.1)	56.2 (56.0–56.1)
	R138G	178.4 ± 4.6 *	41.6 (40.4–44.6)	52.6 (52.5–52.7)	33.2 ± 0.2 *	–	–
	M263V	72.0 ± 1.3 *	39.7 (39.5–39.8)	53.2 (53.1–53.3)	21.9 ± 2.0	–	56.2 (56.1–56.3)
	V400M	141.7 ± 5.4 *	43.3 (43.2–43.5)	50.7 (50.6–50.8)	58.2 ± 0.4 *	48.0 (47.0–49.3)	55.6 (55.5–55.6)
	E414K	96.3 ± 4.4 *	35.9 (35.8–36.0)	52.2 (52.1–52.3)	59.4 ± 0.9 *	–	55.7 (55.7–55.8)
	A421V	165.0 ± 2.6 *	39.7 (39.0–40.6)	46.3 (46.3–46.4)	47.4 ± 0.9 *	47.0 (45.8–51.1)	56.0 (55.9–56.2)
	A433E	72.2 ± 4.8 *	44.6 (44.6–44.7)	45.0 (44.8–45.1)	22.1 ±0.2	47.7 (47.2–48.4)	55.8 (55.7–55.9)
	A433V #	9.3 ± 0.8	–	–	27.0 ± 0.2 *	–	–
	WT #	8.8 ± 0.4	–	–	7.9 ± 0.4	–	–

The protein domains are marked in grey, pink, and blue for easy association with the αDN, βD, and αDC domains, respectively. αDN, N-terminal alpha-helical domain; βD, beta-sheet domain; αDC, C-terminal alpha-helical domain; AU, arbitrary units; F.I., fluorescence intensity; GCDH, glutaryl-CoA dehydrogenase; RALS, right-angle light scattering; TSA, thermal shift assay; Tm_1/2_, first melting transition temperature (lower); Tm_2/3_, second melting transition temperature (higher); WT, wild type. # Protein analyzed in purification buffer containing 10% glycerol (see materials and methods). * *p* < 0.0001 versus wild type, *n* = 8 (TSA) or *n* = 9 (RALS).

## Data Availability

Data are contained within the article.

## Supplementary Materials

The following supporting information can be downloaded at: https://www.mdpi.com/article/10.3390/ijms241713158/s1, Table S1: GCDH mutations and associated frequency and activities.; Table S2: Correlation matrix of *p*-values obtained by pairwise comparison of GCDH variant molecular phenotypes.
