# Supplementary material for: Glutaryl-CoA Dehydrogenase Misfolding in Glutaric Acidemia Type 1
Source: Int J Mol Sci. 2023 Aug 24;24(17):13158. doi: 10.3390/ijms241713158 (PMC10487539; doi:10.3390/ijms241713158)
Supplement: Supplementary file 1 [file ijms-24-13158-s001.zip › ijms-2515464 - Supp Figures.docx]

**Supplementary Material - Figures**


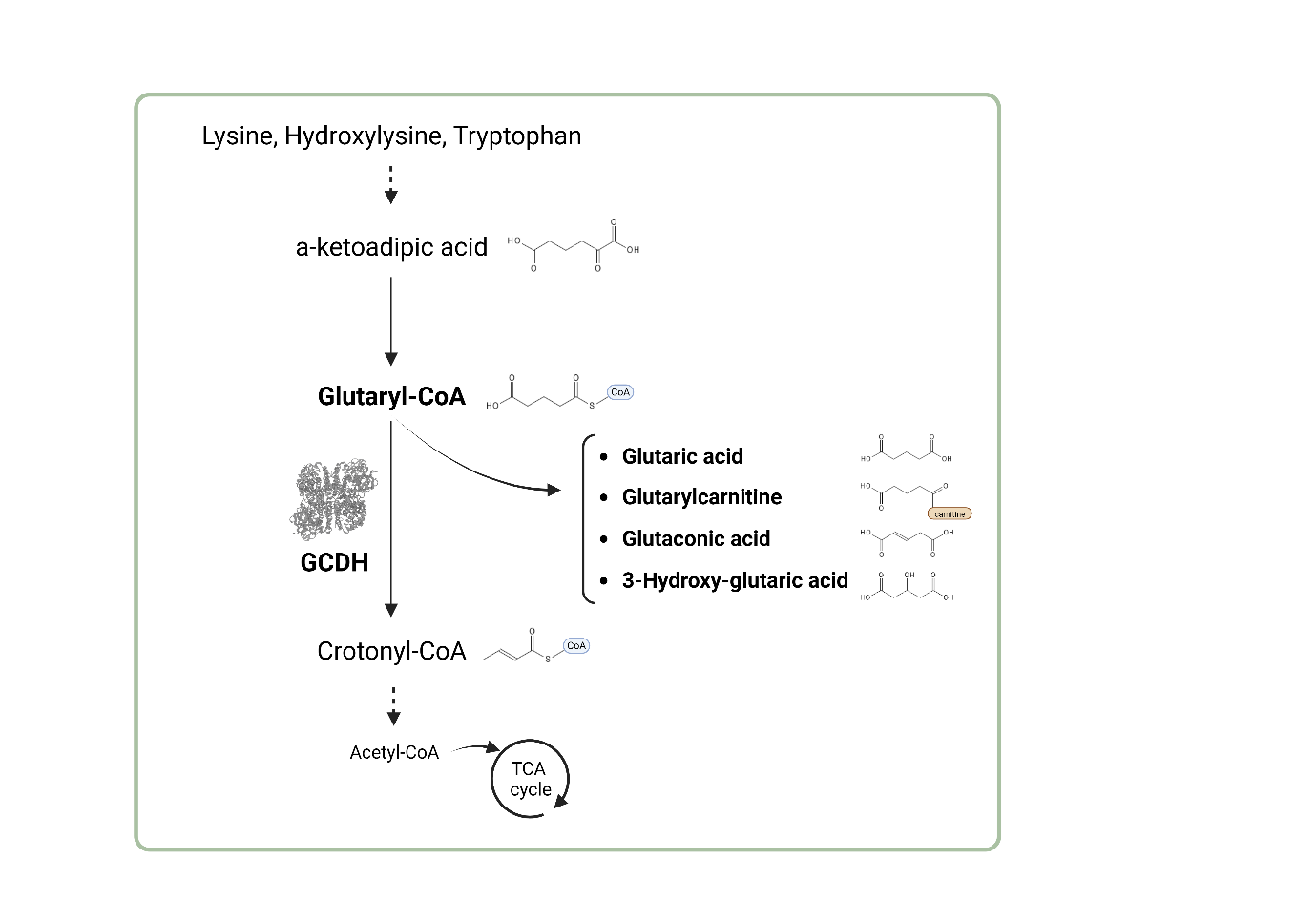


**Figure S1.** Summary representation of glutaryl-CoA dehydrogenase (GCDH) reaction pathway. Chemical structures are shown on the right of the main metabolites involved in GCDH reaction and those increased in glutaric acidemia type I (GA1; bold). This includes the metabolites directly involved in GCDH reaction and the main derivatives found increased in GA1 (glutaric acid, glutarylcarnitine, glutaconic acid, and 3-hydroxyglutaric acid). Dashed arrows represent metabolic reactions that precede and follow GCDH’s reaction.
